# Eating disorder-related electrolyte abnormalities and adverse outcomes: A systematic review and meta-analysis

**DOI:** 10.1371/journal.pone.0349826

**Published:** 2026-06-01

**Authors:** Deena Fremont, Amos Buh, Claire Hoar-Stephens, Nandini Biyani, Rohan Kiska, Stephen G. Fung, Shaafi Mahbub, Muhammad Zameer, Nicholas Fabiano, Marco Solmi, Maya Gibb, Rachel Kang, Maria Salman, Kathryn Lee, Benjamin Milone, Sarah Syed, Shan Dhaliwal, Ayub Akbari, Pierre A. Brown, Manish M. Sood, Gregory L. Hundemer

**Affiliations:** 1 Ottawa Hospital Research Institute, Ottawa, Ontario, Canada; 2 Faculty of Health Sciences, University of Ottawa, Ottawa, Ontario, Canada; 3 Faculty of Science, Carleton University, Ottawa, Ontario, Canada; 4 Bruyère Research Institute, Ottawa, Ontario, Canada; 5 Department of Psychiatry, University of Ottawa, Ottawa, Ontario, Canada; 6 Department of Mental Health, The Ottawa Hospital, Ottawa, Ontario, Canada; 7 Department of Child and Adolescent Psychiatry, Charité Universitätsmedizin Berlin, Berlin, Germany; 8 Faculty of Medicine, University of Ottawa, Ottawa, Ontario, Canada; 9 Faculty of Science, McMaster University, Hamilton, Ontario, Canada; 10 Department of Medicine, Division of Nephrology, University of Ottawa and the Ottawa Hospital, Ottawa, Ontario, Canada; Federal University of Minas Gerais: Universidade Federal de Minas Gerais, BRAZIL

## Abstract

**Objective:**

The aim of this study is to describe the association between electrolyte abnormalities and adverse clinical outcomes, as well as to estimate the prevalence of these abnormalities in individuals with eating disorders.

**Design:**

Preferred Reporting Items for Systematic Reviews and Meta-analyses (PRISMA) 2020-compliant systematic review searching Ovid MEDLINE, EMBASE, and PsycINFO databases from January 2000 to February 2025 was conducted.

**Methods:**

We included studies with any electrolyte abnormality or clinical adverse outcome among individuals with eating disorders. We conducted two meta-analyses to assess (1) the odds of having an electrolyte abnormality among those with an eating disorder diagnosis compared to healthy controls, and (2) the prevalence of electrolyte abnormalities across eating disorder diagnoses.

**Results:**

20 studies incorporating 25,401 individuals were analysed, with most assessing a young female population. Study designs were predominantly retrospective cohort (n = 11) and cross-sectional (n = 5), with few including general population controls (n = 4). Anorexia nervosa was the most common eating disorder studied, with hypokalemia (n = 13 studies), hyponatremia (n = 11 studies), and hypophosphatemia (n = 7 studies) being the most frequently reported electrolyte abnormalities. The most prevalent adverse outcomes included anemia (n = 5 studies) and skeletal conditions (osteoporosis, osteopenia; n = 5 studies). The results from the meta-analyses showed (1) that individuals with eating disorders had significantly higher odds of experiencing electrolyte abnormalities compared to controls (OR = 3.20, 95% CI:1.48–6.94), and (2) varying pooled prevalences of abnormalities, including hypokalemia (15%), hyponatremia (13%), and hypophosphatemia (17%), across studies.

**Conclusion:**

Electrolyte abnormalities are common among individuals with eating disorders and are associated with adverse health outcomes.

**Trial Registration:**

The study was registered with the International Prospective Register of Systematic Reviews (PROSPERO) – (ID CRD42023477497)

## Introduction

Electrolytes are vital to numerous physiological processes, including nerve and muscle function, biochemical reactions, and maintaining fluid and pH balance [1]. Abnormal electrolyte levels, whether elevated or reduced, can disrupt bodily functions, and subsequently lead to life threatening complications, including cardiac arrhythmias and neurologic disturbances [1–3]. Symptoms of electrolyte abnormalities include constipation, nausea and vomiting, fatigue, heart palpitations or arrhythmias, muscle weakness, cramps, numbness, polyuria, headache, confusion, restlessness, and irritability, among others [4].

Electrolyte abnormalities are common among individuals with eating disorders, due to severe food restriction, binge eating, excessive exercise, chronic vomiting, and abuse of laxatives or diuretics [5,6]. Aggressive interventions for eating disorders can trigger refeeding syndrome, which involves the rapid reabsorption of electrolytes into cells, which further exacerbate electrolyte abnormalities [5,7–13]. Due to variations in disordered eating behaviours and treatment approaches, the severity and type of electrolyte abnormalities can vary considerably among individuals with eating disorders [14,15].

While the association between electrolyte abnormalities and specific conditions, such as chronic kidney disease, have been identified in individuals with eating disorders, the full range of adverse clinical events remains unknown [15–17]. As such, we conducted a systematic review to assess the association between electrolyte abnormalities related to eating disorders, and their adverse clinical outcomes.

## Methods

### Study design

This systematic review was developed based on the Preferred Reporting Items for Systematic Reviews and Meta-Analyses Protocols (PRISMA-P) criteria (S1 Table) [18]. The study was registered with the International Prospective Register of Systematic Reviews (PROSPERO) – (ID CRD42023477497). The study protocol has previously been published [19].

### Inclusion/ exclusion criteria

This review included studies assessing individuals of all ages with electrolyte abnormalities in association with having an eating disorder diagnosis, or disordered eating behaviours as per the original study authors definitions. Outcome measures included adverse clinical outcomes, hospitalization, and death. This review excluded studies that only assessed electrolyte abnormalities following inpatient eating disorder treatment (e.g., refeeding syndrome).

### Types of studies

This review included cohort, cross-sectional, and case-control studies. This review excluded case reports and commentaries.

### Search strategy

The literature search was conducted in March 2025. We searched the following databases for articles published between January 2000 and February 2025: Ovid MEDLINE, EMBASE, and PsycINFO. We restricted the search to studies published from the year 2000 onwards to reflect contemporary changes in the diagnosis and treatment of eating disorders [20–22]. We also completed a manual search of the literature, in addition to screening reference lists and articles of included studies. The search strategy was created in consultation with a health sciences librarian (MCD) with expertise in systematic reviews and meta-analysis. The MEDLINE search strategy is provided in the supplement, while the full search strategies for all datasets are available upon request (S2 Table).

### Study screening and selection

All relevant articles generated from the search strategy were imported into Covidence software for screening [23]. Two reviewers independently screened titles and abstracts for inclusion. Articles deemed appropriate as per our inclusion criteria were retrieved and reviewed for final full text inclusion. Conflicts were reviewed by a third reviewer.

### Data extraction

Data was extracted using a standardized data extraction tool from the Joanna Briggs Institute Manual for Evidence Synthesis [24]. The following information was extracted: study author and year, setting and location, study design, population of interest, sample size, intervention, comparison group, outcomes, and association measures. In the event of missing data from a study, the corresponding author was contacted.

### Data synthesis and meta-analyses

We summarized studies according to eating disorder diagnosis, type of electrolyte abnormality, and adverse outcome(s) experienced [24]. We conducted two meta-analyses, which assessed (1) the odds of having an electrolyte abnormality among those with an eating disorder diagnosis compared to healthy controls, and (2) the prevalence of electrolyte abnormalities (specifically hypokalemia, hypomagnesemia, hyponatremia, hypophosphatemia, and metabolic alkalosis) across all eating disorder diagnoses utilizing random effects models [25]. We restricted the prevalence meta-analyses to electrolyte abnormalities with three or more studies reporting on the condition. We calculated odds ratios (ORs) and proportions. Heterogeneity was assessed utilizing both the I^2^ and τ^2^ statistics.

### Critical appraisal: Methodological quality (JBI)

The methodological quality of the included studies was assessed using a standard critical appraisal tool from the Joanna Briggs Institute (JBI) for cohort, cross-sectional, and case-control studies by two independent reviewers. Based on previous systematic reviews, we assessed methodological quality results in the following categories: Studies with scores higher than 70% were considered high quality, studies with scores between 50–70% were considered moderate quality, and studies with scores below 50% were considered low quality [26].

## Results

**Fig 1:**
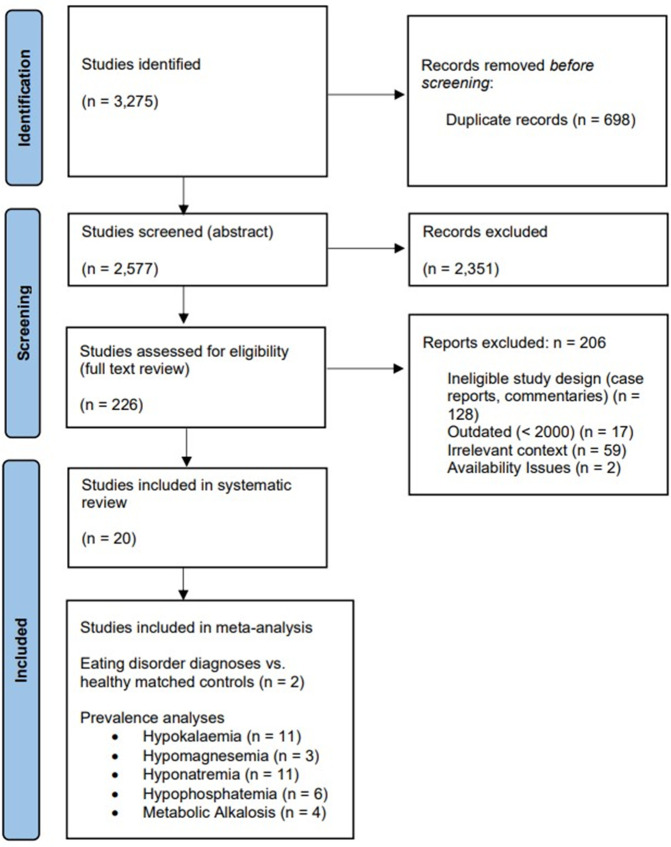
PRISMA flowchart of study selection process.

Our search of Ovid MEDLINE, EMBASE, and PsycINFO yielded 3,275 studies. Following this, 698 duplicate studies were removed prior to abstract screening. During title/abstract screening, a further 2,351 studies were removed. The remaining 226 studies proceeded to full text review. A total of 206 studies were excluded for various reasons; 128 had an ineligible study design (i.e., case reports, commentaries), 17 were published before the year 2000, 59 were deemed to be of irrelevant context, and 2 due to full text availability issues (S3 Table). The remaining 20 [27–46] studies were included in this systematic review, with a subset of 15 studies being further used in meta-analysis [27–31,33–37,39,42–44,46] (**Fig 1**).

### Study characteristics

Across the 20 included studies, 25,401 cases of eating disorders were identified (**Table 1**). Eating disorder cases were defined according to the original investigators’ criteria. Only four of these studies compared individuals with eating disorders to healthy controls, totalling 28,157 individuals [27,32,36,38]. The majority of studies were retrospective cohort (n = 11) [31,33–35,37,39–41,43–45] and cross sectional (n = 5) [28,29,32,38,46]. Studies were predominantly conducted in the United States of America (n = 9) [28,29,31,40–45], Israel (n = 3) [32,34,36], France (n = 3) [35,39,46], and Canada (n = 2) [27,37]. Mean age among both individuals with eating disorders, and if applicable healthy controls, varied (ranging from 13.9 ± 3.3 [40] to 29.8 ± 11.42 years [42] and 15.8 ± 0.8 [32] to 28 ± 19 years [27], respectively). Nearly half (n = 8) [28–30,32,34,36,38,46] of studies limited their analysis to a female only population. Among the remaining 12 studies that did assess both sexes [27,31,33,35,37,39–45], the prevalence of male representation was consistently low, with the prevalence ranging from 1.4% [44] to 29.4% [40] of the total sample. Detailed characteristics of the studies are summarized in **Table 1**.

**Table 1:** Characteristics of included studies.

Study (first author, year)	Design	Setting	Sample			Comparator Group (Healthy controls)		
			Description including sociodemographic characteristics (n)	Age, years (mean ± SD)	Sex, n (%)	Description including sociodemographic characteristics (n)	Age, years (mean ± SD)	Sex
Caregaro, 2005	Prospective cohort study	Hospital, Milan, Italy	N = 14 patients with AN (admitted for serve malnutrition) Race and/or Ethnicity: NR SES: NR	22.64 ± 4.06	Females = 14 (100.0) Males = 0 (0.0)	N/A		
Mehler, 2018	Retrospective cohort study	Eating Recovery Center, Denver, Colorado, United States of America	N = 1,026 inpatients with ED’s (chart review) Race and/or Ethnicity: Caucasian (93.2%) SES: NR	28.1 ± 10.1	Females = 960 (93.6) Males = 66 (6.4)	N/A		
Miller, 2005	Cross-sectional cohort study	Hospital, in Massachusetts, United States of America	N = 214 women (17 and 45 years) with AN (recruited via community-based referrals) Race and/or Ethnicity: NR SES: NR	25.0 ± 6.4	Females = 214 (100.0) Males = 0 (0.0)	N/A		
Tannhauser, 2001	Cross-sectional cohort study	Outpatient medical clinic, Israel	N = 45 female Jewish Israeli outpatients with primary AN of less than 30 months’ duration and no previous hospitalization Race and/or Ethnicity: Jewish (100%) SES: NR	16.3 ± 1.8	Females = 45 (100.0) Males = 0 (0.0)	N = 156 healthy schoolgirl controls (matched on age, religious practices, and socioeconomic status) Race and/or Ethnicity: Jewish (100%) SES: Matched on SES, but not described	15.8 ± 0.8	Females = 156 (100.0) Males = 0 (0.0)
Whitelaw, 2014	Retrospective cohort study	Children’s hospital, Australia	N = 99 (comprised of n = 73 patients with AN + n = 26 with EDNOS admitted to hospital) Race and/or Ethnicity: NR SES: NR	15.2 ± 1.3	Females = 86 (87.0) Males = 13 (13.0)	N/A		
Levy-Shraga, 2016	Retrospective cohort study	Hospital, Tel Aviv, Israel	N = 174 adolescent females (mean age 15.7 ± 1.8 y) hospitalized due to AN Race and/or Ethnicity: NR SES: NR	15.7 ± 1.8	Females = 174 (100.0) Males = 0 (0.0)	N/A		
Hundemer, 2022	Matched (4:1) case-control study	Ontario, Canada	N = 6,970 Ontario residents aged 13 or older diagnosed with an ED Race and/or Ethnicity: NR SES: Neighbourhood income quintiles (1^st^: 20.1%, 2^nd^: 18%, 3^rd^: 18.6%, 4^th^: 19.3%, 23.3%)	28 ± 19	Females = 6,075 (87.2) Males = 895 (12.8)	N = 27,878 age- and sex-matched residents without an ED Race and/or Ethnicity: NR SES: Neighbourhood income quintiles (1^st^: 19%, 2^nd^: 19.2%, 3^rd^: 20.5%, 4^th^: 20.4%, 20.5%)	28 ± 19	Females = 24,300 (87.2) Males = 3,578 (12.8)
Guinhut, 2021	Retrospective cohort study	Hospital, Garches, France	N = 354 AN patients hospitalized for extreme malnutrition Race and/or Ethnicity: NR SES: Qualification level > 4 years (26.1%), Marital status (couple/married: 20.3%, divorced: 7.1%, single: 71.5%, widow: 1.1%)	28.7 ± 10.7	Female = 339 (95.8) Male = 15 (4.2)	N/A		
Lawson, 2012	Cross-sectional cohort study	Hospital, Massachusetts, United States of America	N = 404 women (18–54 years of age) with AN Race and/or Ethnicity: NR SES: NR	25.6 ± 0.3	Female = 404 (100.0) Males = 0 (0.0)	N/A		
Solmi, 2024	Retrospective cohort study	Ontario, Canada	N = 6,163 patients (aged 13 +) with an ED and an electrolyte measure within 1 year since diagnosis Race and/or Ethnicity: NR SES: Neighbourhood income quintiles (1^st^: 19.1%, 2^nd^: 17.6%, 3^rd^: 18.4%, 4^th^: 20.6%, 23.7%)	26.8 ± 17.5	Females = 5,456 (88.5) Males = 707 (11.5)	N/A		
Wolfe, 2001	Case-control study	Hospital, Israel	N = 74 female non-hospitalized participants with BN Race and/or Ethnicity: NR SES: NR	24 ± 5	Females = 74 (100.0) Males = 0 (0.0)	N = 110 female volunteers with no history of a psychiatric disorder (sex and weight matched) Race and/or Ethnicity: NR SES: NR	23 ± 4	Females = 110 (100.0) Males = 0 (0.0)
Zepf, 2017	Cross-sectional cohort study	Hospital, Frankfurt am Main, Germany	N = 23 total BN females: Separated into acutely ill (N = 11) and remitted (N = 12) adolescent and young adult patients with BN Race and/or Ethnicity: NR SES: NR	20.08 ± 2.85 (BN acute) 24.80 ± 2.48 (BN remitted)	Females = 23 (100.0) Males = 0 (0.0)	N = 13 healthy controls (similar age) Race and/or Ethnicity: NR SES: NR	25.32 ± 2.58	Females = 13 (100.0) Males = 0 (0.0)
Abbas, 2025	Retrospective cohort study	Pediatric hospital, Lyon, France	N = 30 hospitalized children (< 18 years) with AN severely undernourished who benefitted from a pediatric enteral refeeding protocol elaborated for the study Race and/or Ethnicity: NR SES: NR	14.0 ± 2.49	Females = 27 (90) Males = 3 (10)	N/A		
Downey, 2024	Retrospective cohort study	Medicaid claims data for service episodes, California, United States of America	N = 8,075 youth (aged 7–18) who had at least one service episode were found to have an ED Race and/or Ethnicity: NR SES: NR	13.9 ± 3.3	Females = 5,701 (70.6) Males = 2,374 (29.4)	N/A		
Dunbar, 2023	Retrospective cohort study	Hospital, United States of America	N = 81 hospitalized patients (aged 11–26 years) with restrictive eating disorder at < 75% treatment goal weight Race and/or Ethnicity: Race (Caucasian: 86.4%, Asian: 4.9%, unspecified: 8.6%), Ethnicity (non-Hispanic/Latino: 92.6%, Hispanic/Latino: 6.2%, unspecified: 1.2%) SES: NR	17.8 ± 3.5 and 17.0 ± 3.5	Females = 73 (90.1) Males = 7 (8.6) Transgender = 1 (1.2)	N/A		
Kells, 2023	Case-control study	Centre for eating disorders and severe malnutrition, Hospital, Denver, United States of America	N = 307 patients admitted to hospital with a diagnosis of AN or ARFID (avoidant/restrictive food intake disorder) Race and/or Ethnicity: NR SES: NR	29.8 ± 11.42	Females = 280 (91.2) Males = 27 (8.8)	N/A		
Leach, 2024	Retrospective cohort study	Centre for eating disorders and severe malnutrition, Hospital, Denver, United States of America	N = 545 patients with severe AN or avoidant restrictive food intake disorder hospitalized in a medical stabilization unit Race and/or Ethnicity: Race (Asian: 1%, Black/African American: 1%, unknown: 4%, Native Hawaiian: < 1%, white/Caucasian: 92%, other: 2%), ethnicity (Hispanic, Latinx, or Spanish origin: 1%, Non- Hispanic, Latinx, or Spanish origin: 94%, unknown: 5%) SES: NR	31.8 ± 12.3	Female = 490 (90) Males = 55 (10)	N/A		
Manwaring, 2024	Retrospective cohort study	Hospital, United States of America	N = 69 adult patients with a diagnosis of atypical AN admitted to hospital unit Race and/or Ethnicity: American Indian/Native American/Alaskan Native (1.45%), Black (1.45%), multiracial (1.45%), white (95.65%) SES: NR	26.61 ± 8.69	Females = 68 (98.6) Males = 1 (1.4)	N/A		
Nagata, 2022	Retrospective cohort study	Hospital, San Francisco, United States of America	N = 537 patients (9–25 years) with an initial hospitalization for the medical management of an eating disorder and a plasma zinc concentration measurement Race and/or Ethnicity: Non-Hispanic White (60.15%), Hispanic (16.20%), Asian or Native Hawaiian and Other Pacific Islanders (7.64%), Multiracial (5.96%), Other (5.03%), unknown/declined (3.17%), Non-Hispanic Black or African American (1.86%) SES: NR	15.98 ± 2.81	Females = 450 (83.8) Males = 87 (16.2)	N/A		
Stheneur, 2024	Cross-sectional cohort study	Multi-centre (11 sites) Hospital units, France	N = 197 hospitalized female participants (aged 13–65) with full syndrome or sub-threshold AN that have BMI < 15 kg/m2 and/or sudden and rapid weight loss Race and/or Ethnicity: NR SES: NR	20.74 ± 6.5	Females = 197 (100.0) Males = 0 (0.0)	N/A		

Abbreviations: SES: Socioeconomic status, NR: Not Reported, SD; Standard Deviation, AN; Anorexia Nervosa, ED; Eating Disorder, BN; Bulimia Nervosa, EDNOS; Eating Disorders Not Otherwise Specified.

### Outcomes

#### Types of eating disorders.

Eating disorders included anorexia nervosa (AN) (85%) [27–35,37,39,40,42–46], bulimia nervosa (BN) (25%) [27,31,36–38,40,45], avoidant restrictive food intake disorder (ARFID) (20%) [41,43,45], and eating disorders not otherwise specified (EDNOS) (30%) (**Table 2**) [27,31,33,37,40,45]. Most studies focused on AN or associated AN-subtypes. Subtypes of AN assessed included restricting AN (AN-R) [28–35,42,43,45,46], purging AN (AN-P) [28,34], and binge eating/purging AN (AN-BP) [29–31,35,42,43,46]. Studies were predominantly conducted across various types or subtypes of eating disorder diagnoses (n = 14). Of the remaining six studies, two studies focused exclusively on individuals with AN [39,44], one study on individuals with AN-R [32], two studies on individuals with BN [36,38], and one study on individuals with ARFID [41].

**Table 2:** Type of eating disorder(s), electrolyte abnormalities, and adverse clinical outcomes among selected studies.

Study (first author, year)	Type/Subtype(s) of Eating Disorder(s)	Type(s) of Electrolyte Abnormalities	Adverse Clinical Outcomes
Caregaro, 2005	AN-R, AN-BP	• Hypokalemia on admission (14.3%; 2/14) • Hyponatremia on admission (21.4%; 3/14)	• Plasma volume depletion on admission (64%) • Hyponatremia was associated with hypovolemia (hypovolemic hyponatremia) (21.4%) • Anemia (42.9%)
Mehler, 2018	AN-R, AN-BP, BN, EDNOS	Whole Sample • Hyponatremia (14%; 144/1026) • Hypokalemia (25.8%; 265/1026) • Metabolic alkalosis (16.6%; 170/1026) ------------------ AN-R • Hyponatremia (16.0%) • Hypokalemia (14.2%) • Severe metabolic alkalosis (1.3%) AN-BP • Hyponatremia (17.1%) • Hypokalemia (42.4%) • Severe metabolic alkalosis (11.8%) BN • Hyponatremia (8.5%) • Hypokalemia (26.2%) • Severe metabolic alkalosis (7.7%) EDNOS • Hyponatremia (11.8%) • Hypokalemia (22.5%) • Severe metabolic alkalosis (9.8%)	Whole Sample • Osteoporosis (23.9%) • Osteopenia (30.7%) • Tachycardia (3.1%) • Bradycardia (35.2%) ---------------- AN-R • Osteoporosis (34.3%) • Osteopenia (25.9%) • Tachycardia (1.9%) • Bradycardia (48.4%) AN-BP • Osteoporosis (21.1%) • Osteopenia (34.8%) • Tachycardia (3.5%) • Bradycardia (38.6%) BN • Osteoporosis (8.9%) • Osteopenia (34.1%) • Tachycardia (4.2%) • Bradycardia (18.4%) EDNOS • Osteoporosis (4.0%) • Osteopenia (33.3%) • Tachycardia (4.0%) • Bradycardia (15.2%)
Miller, 2005	AN-R, AN-P	• Hyponatremia (19.7%; 42/214) • Hypokalemia (19.7%; 42/214)	• Anemia (38.6%) • Leukocytopenia (34.4%) • Bradycardia (41.3%) • Hypotension (16.1%) • Hypothermia (22.4%) • Elevation of Alanine Aminotransferase (ALT) Concentration (12.2%) • Osteopenia (51.7%) • Osteoporosis (34.6%) • Primary Amenorrhea (14.8%)
Tannhauser, 2001	AN-R	• All patients were zinc deficient upon admission (100%): Zinc deficiency (56.3 ± 10.2 mcg/dl) • Twenty-four-hour urinary zinc excretion was in the deficient range (140.3 ± 86.2 mcg/24 hr)	None reported
Whitelaw, 2014	AN-R, EDNOS	Whole Sample • Hypophosphatemia (40.4%; 40/99) • Hypomagnesemia (9.1%; 9/99) • Hypokalemia (4.0%; 4/73) -------------------------------- AN • Hypophosphatemia (41%) • Hypomagnesemia (11%) • Hypokalemia (5%) EDNOS • Hypophosphatemia (38%) • Hypomagnesemia (4%) • Hypokalemia (0%)	AN • Lowest pulse rate (Mean + SD) = 45.1 bmp (9.9) • Lowest systolic blood pressure (Median IQR) = 84 (80–90) EDNOS • Lowest pulse rate (Mean + SD) = 47.1 bmp (13.7) • Lowest systolic blood pressure (Median IQR) = 91 (87–94)
Levy-Shraga, 2016	AN-R, AN-P	• Hyponatremia upon admission (10.9%; 64/174) • Hyponatremia in the year preceding BMD measurement (36.8%) • Participants with hyponatremia had slightly lower levels of calcium and phosphorus compared with those with no documented hyponatremia	• Participants with hyponatremia had a significantly lower lumbar spine BMD z-score (−1.8 ± 1.2 versus −1.3 ± 1.2, P = 0.01) compared with those with no documentation of hyponatremia • Participants with hyponatremia also had a lower BMI z-score (−2.6 ± 1.3 versus −2.2 ± 1.2, P = 0.04) compared with those with no documentation of hyponatremia Comorbidities • Depressive disorder (52.9%) • Anxiety disorder (9.2%) • Obsessive compulsive disorder (12.1%) • Attention deficit hyperactivity disorder (6.3%)
Hundemer, 2022	AN, BN, EDNOS	• 18.4% (1282/6970) of individuals with an eating disorder had a preceding electrolyte abnormality vs 7.5% (2091/27878) of individuals without an eating disorder Electrolyte abnormalities were more common in individuals with an eating disorder than compared to controls without: • Hypokalemia (12.1% vs. 4.6%; 878/6970) • Hyperkalemia (2.8% vs. 1.3%) • Hyponatremia (2.5% vs. 0.4%; 174/6970) • Hypernatremia (0.3% vs. 0.1%) • Hypomagnesemia (0.2% vs. 0.1%; 14/6970) • Hypophosphatemia (1.9% vs. 0.4%; 132/6970) • Metabolic acidosis (2.3% vs. 1.2%) • Metabolic alkalosis (1.4% vs. 0.4%; 98/6970)	Comorbidities (cases vs. controls) • Hospitalization within prior 2-years (26.8% vs. 11.3%) • Prior outpatient psychiatry visit (53.0% vs. 13.1%) • Anxiety (19.7% vs. 4.7%) • Asthma (17.3% vs 13.5%) • Chronic kidney disease (2.9% vs. 1.4%) • Chronic obstructive pulmonary disease (4.6% vs. 2.3%) • Congestive heart failure (3.1% vs. 1.4%) • Depression (19.3% vs. 3.4%) • Diabetes (9.1% vs. 7.3%) • Inflammatory bowel disease (1.8% vs. 1.1%) • Liver disease (4.2% vs. 2.3%) • Myocardial infarction (0.5% vs. 0.4%) • Personality disorder (4.3% vs. 0.4%) • Substance abuse (8.1% vs. 1.9%)
Guinhut, 2021	AN-R, AN-BP	• Zinc deficiency (56.8%) • Hypernatremia (1.1%) • Hyponatremia (22.3%; 79/354) • Hypophosphatemia (26.0%; 92/354) • Hypokalemia (39.5%; 140/354) • Dysnatremia (24.3%)	• Anemia (79%) • Neutropenia (53.9%) • Hypertransaminasemia (53.7%) • Osteoporosis (46.3%) • Hypoglycemia (13.8%) • Infectious complications (24.3%) • Cardiac dysfunction (7.1%) • Gelatinous bone marrow transformation (6.5%) • ICU referrals (10%) • Mortality (1.4%) Comorbidities: • Psychiatric comorbidities (47.5%) • Associated somatic comorbidities (19.8%)
Lawson, 2012	AN-R, AN-BP	• Hyponatremia (3.0%; 12/404)	• Women with hyponatremia were older, had lower BMI, and reported greater duration of anorexia nervosa than those without hyponatremia • A greater percentage of the women with hyponatremia reported current psychiatric medication use than those without (83% vs. 53%) • Amenorrhea was higher among women with hyponatremia (75%) vs without (64%) • Those with Hyponatremia (defined as plasma sodium < 135 mmol/L) had lower Bone Mineral Density, T- and Z-scores at the anterior-posterior spine and total hip compared to those with sodium levels at or above 140 mmol/L
Wolfe, 2001	BN	• Hyponatremia in those with BN vs. controls (6.8% (5/74) vs. 0.9%) • Hypokalemia in those with BN vs. controls (5.6% (4/74) vs. 0.0%) • Hypochloremia in those with BN vs controls (8.1% vs. 0.0%) ◦ Overall BN vs controls (6/74 vs. 1/110) • Patients who reported more than 14 episodes per week of self-induced vomiting had a particularly high frequency of hypokalemia (x2 = 9.84, p < .01) and hypochloremia (x2 = 7.55, p < .02) • Although not significantly related to symptom frequency by categorical analysis, serum TCO2 showed a positive correlation with frequency of self-induced vomiting (rho = 0.39, p < .001).	• Patients with BN had a lower pulse than controls (58 ± 11 vs. 63 ± 10 beats per minute; z = 3.65, p < .001) • Electrocardiograms were available for 4/5 of patients with BN with low K+ and one control with low K + : Slight electrocardiogram abnormalities (borderline QTc prolongations) were present in 2 patients with BN
Solmi, 2024	AN, BN, EDNOS	32.2% of patients had any electrolyte abnormality within 1-year of ED diagnosis. Of those with any electrolyte abnormality, the types are as follows: • Hypokalemia (50.0%; 3082/6163) • Hyponatremia (37.8%; 2330/6163) • Hypernatremia (21.1%) • Hyperkalemia (6.6%) • Metabolic acidosis (8.7%) • Metabolic alkalosis (8.6%; 530/6163)	Outcomes among those with an eating disorder that have an electrolyte abnormality vs. those with an eating disorder without electrolyte abnormalities • Hospitalization (60.5% vs. 47.4%) • Acute kidney injury (10.4% vs. 3%) • Chronic kidney disease (12.3% vs. 4.3%) • Bone fracture (7.0% vs. 4.0%) • Bowel obstruction (3.6% vs. 1.4%) • Death (15.7% vs. 5.6%)
Zepf, 2017	BN	• Acutely ill patients with BN had significantly higher Zinc levels when compared with controls (t = 2.152, P = 0.043, df = 1, 22) • Remitted patients with BN also had significantly higher serum Zinc concentrations when compared to controls (t = 5.052, P = 0.000, df = 1, 23) • There was no statistically significant difference in Zinc concentrations between acutely ill and remitted patients with BN (t = 1.089, P = not significant [ns], df = 1, 21).	• Acutely ill patients with BN tended to show higher leptin concentrations when compared to healthy controls (t = 1.879 P = 0.074, df = 1, 22), though this did not reach conventional statistical significance • There was no difference in leptin concentrations between controls and remitted patients with BN (t = 1.677, P = ns, df = 1, 23), as well as between acutely ill and remitted patients with BN (t = 0.118, P = ns, df = 1, 21).
Abbas, 2025	AN	• Hypophosphatemia (16.6%; 5/30) • Hypokalemia (0%; 0/30) • Hypomagnesemia (0%; 0/30)	Outcomes among people with AN • Hepatic Cytolysis (20.0%) • Hypoglycemia (37.9%) • Hyperglycemia (0.0%) • Anemia (3.4%) • Lymphocytopenia (3.6%)
Downey, 2024	AN, BN, Other specified feeding or eating disorder, other ED, and unspecified ED	Whole Sample: Electrolyte abnormality (defined as hypophosphatemia, hypomagnesemia, and/or hypokalemia) was 2.4% ------------------------- Electrolyte abnormality (defined as hypophosphatemia, hypomagnesemia, and/or hypokalemia), among subgroups of ED’s: • AN (3.9%) • BN (2.4%) • Other specified feeding or eating disorder (1.9%) • Other ED (1.1%) • Unspecified ED (2.2%)	Whole Sample: • Medical instability (7.1%) • Cardiovascular (4.3%) • Hypotension (0.9%) • Orthostasis (1.0%) • Malnutrition (2.5%) • Gastrointestinal (3.0%) • Hematological (11.9%) • Endocrinologic (2.9%) • Renal (3.0%) • Skeletal (5.4%) ---------------------------- AN • Medical instability (16.3%) • Cardiovascular (11.6%) • Hypotension (2.6%) • Orthostasis (2.3%) • Malnutrition (7.9%) • Gastrointestinal (2.8%) • Hematological (13.6%) • Endocrinologic (6.7%) • Renal (0.9%) • Skeletal (5.3%) BN • Medical instability (10.7) • Cardiovascular (3.2%) • Hypotension (0.5%) • Orthostasis (0.5%) • Malnutrition (1.8%) • Gastrointestinal (3.7%) • Hematological (10.9%) • Endocrinologic (2.3%) • Renal (0.7%) • Skeletal (4.0%) Other specified feeding or eating disorder • Medical instability (4.5%) • Cardiovascular (2.4%) • Hypotension (0.6%) • Orthostasis (0.3%) • Malnutrition (1.9%) • Gastrointestinal (3.2%) • Hematological (10.7%) • Endocrinologic (1.4%) • Renal (9.4%) • Skeletal (8.1%) Other ED • Medical instability (1.7%) • Cardiovascular (0.7%) • Hypotension (0.0%) • Orthostasis (0.0%) • Malnutrition (0.2%) • Gastrointestinal (2.0%) • Hematological (21.0%) • Endocrinologic (1.3%) • Renal (7.9%) • Skeletal (7.2%)
			Unspecified ED• Medical instability (6.3%)• Cardiovascular (3.6%)• Hypotension (0.7%)• Orthostasis (1.1%)• Malnutrition (3.6%)• Gastrointestinal (3.1%)• Hematological (11.1%)• Endocrinologic (2.6%)• Renal (1.4%)• Skeletal (4.5%)
Dunbar, 2023	Restrictive Eating Disorder	Whole Sample • Hypophosphatemia, hypokalemia, and/or hypomagnesemia (29.6%)	Comorbid psychiatric conditions were present in 79.3% of participants: • Anxiety (94%) • Mood disorder (56%) • Developmental disorders (12%) Medical comorbidities included: • Gastrointestinal (19.0%) • Pulmonary (9.5%) • Neurological (7.8%) • Endocrine (6.3%) • Haematological (4.8%) • Cardiac (3.2%)
Kells, 2023	AN, AN-R, AN-BP, ARFID	Whole Sample • Hypophosphatemia (35.5%; 109/307) -------------- AN • Hypophosphatemia (33.3%) ARFID • Hypophosphatemia (44.8%) AN-R • Hypophosphatemia (30.1%) AN-BP • Hypophosphatemia (36.0%)	None reported
Leach, 2024	AN-R, AN-BP, ARFID	Whole Sample: • Hypokalemia (39%; 213/545) • Metabolic alkalosis (53%; 289/545) • Hyponatremia (25%; 136/545) -------------- AN-R • Hypokalemia (29%) • Alkalosis (41%) • Hyponatremia (not specified in breakdown) AN-BP • Hypokalemia (57%) • Metabolic alkalosis (46%) • Hyponatremia (not specified in breakdown) ARFID • Hypokalemia (14%) • Metabolic alkalosis (14%) • Hyponatremia (not specified in breakdown)	Whole Sample • Pseudo-Bartter's Edema (33.6%) -------------- AN-R • Pseudo-Bartter's Edema (12%) AN-BP • Pseudo-Bartter's Edema (18%) ARFID • Pseudo-Bartter's Edema (4%)
Manwaring, 2024	AN	• Hypophosphatemia (11.59%; 8/69)	• Bradycardia (14.49%) • Osteopenia or Osteoporosis (4.3%)
Nagata, 2024	AN, ARFID, BN, Binge-eating disorder, Other specified feeding and eating disorder, Unspecified Feeding and Eating Disorder, Other	• Zinc deficiency (24.77%)	• Anemia (22.67%)
Stheneur, 2024	AN-R, AN-BP	• Hyponatremia (3.0%; 6/197) • Hypokalemia (2.0%; 4/197) • Hypochloremia (4.6%)	None reported

AN = anorexia nervosa, AN-R = restricting anorexia nervosa, AN-BP = binge eating/purging anorexia nervosa, AN-P = purging anorexia nervosa, BN = Bulimia Nervosa, EDNOS = eating disorder not otherwise specified, ARFID = avoidant restrictive food intake disorder, SD = Standard Deviation.

#### Electrolyte abnormalities.

Eating disorders were found to be associated with various electrolyte abnormalities (**Table 2**). The most common electrolyte abnormalities included hypokalemia [10,27,28,30,33,35–37,39–41,43,46] (ranging from 0% [39] to 50% [37]), hyponatremia [10,27–30,34–37,43,46] (ranging from 3% [29,46] to 37.8% [37]), and hypophosphatemia [27,33,35,39,40,42,44] (ranging from 1.9% [27] to 40.4% [33]). Less commonly, some studies reported on outcomes such as hypomagnesemia [27,33,39–41] (ranging from 0% [39] to 9.1% [33]), hypochloremia [36,46] (ranging from 4.6% [46] to 8.1% [36]), and metabolic alkalosis [10,27,37,43] (ranging from 1.4% [27] to 53.3% [43]). Results on zinc deficiency among people with an eating disorder diagnosis varied [32,35,38,45], with rates ranging from 24.77% [45] to 100% [32] of samples.

Several studies assessed electrolyte abnormities by types and subtypes of eating disorder diagnoses (**Table 2**). Studies predominately reported AN-BP as the eating disorder subtype with the highest prevalence of electrolyte abnormalities, including hypokalemia [10,43], hyponatremia [10], and metabolic acidosis [10,43]. However, for conditions such as hypophosphatemia [33,40] and hypomagnesemia [33], AN is typically identified as the eating disorder diagnosis with the highest prevalence of these abnormalities. Finally, two studies assessed zinc deficiency only in a single type of eating disorder diagnosis. Tannhauser and colleagues found that at hospitalization admission, all patients with AN-R were zinc deficient [32]. However, Zepf and colleagues reported different results among individuals with BN [38]. Their study found that individuals with BN had elevated zinc concentrations when compared to healthy controls [38].

#### Clinical outcomes.

Various adverse clinical outcomes were prevalent across all eating disorder diagnoses (**Table 2**). The most common adverse outcomes assessed included anemia [28,30,35,39,45] (ranging from 3.4% [39] to 79.0% [35]) and skeletal conditions [10,28,35,40,44] (including osteoporosis and osteopenia) (ranging from 4.3% [44] to 51.5% [28]). Other studies focused on different clinical outcomes, including bradycardia [10,44] (14.49% [44] and 35.2% [10]), hypotension [28,40] (0.9% [40] and 16.1% [28]), chronic kidney disease [27,37] (2.9% [27] and 12.3% [37]), Pseudo-Bartter's Edema [43] (33.6% [43]), mortality [35,37] (1.4% [35] and 15.7% [37]), and gastrointestinal [40,41] (3.0% [40] and 19.0% [41]), haematological [40,41] (4.8% [41] and 11.9% [45]), and endocrinological [40,41] (2.9% [40] and 6.3% [41]) conditions. Three studies assessed mental health comorbidities [27,34,41], including anxiety [27,34,41] (9.2% [34], 19.7% [27], 94% [41]), and depression [27,34] (19.3% [27] and 52.9% [34]). Two studies assessed health service utilization [27,37], reporting the use of hospitalization ranging from 26.8% [27] to 60.5% [37] among those with an eating disorder.

Few studies assessed adverse clinical outcomes by types and subtypes of eating disorder diagnoses (**Table 2**) [10,40,43]. Downey and colleagues reported that those with AN had a higher prevalence of clinical complications or comorbidities, including medical instability (16.3%), hypotension (2.6%), malnutrition (7.9%), cardiovascular (11.6%) and endocrinological (6.7%) complications, when compared to individuals with BN and other specified feeding and eating disorders [40]. In this same study, gastrointestinal complications were the only clinical outcome represented by a higher proportion of BN diagnoses (3.7%) compared to AN [40]. Other studies reported similar results of AN predominance across clinical outcomes, including osteoporosis (AN-R 34.3% [10]), osteopenia (AN-BP 34.8% [10]), bradycardia (AN-R 48.4% [10]), and Pseudo-Bartter's Edema (AN-BP 18.0% [43]). In a study by Mehler and colleagues, tachycardia was the only clinical outcome represented by a higher proportion of BN diagnoses (3.7%) than compared to AN [10].

Lastly, three studies assessed whether those with an eating disorder and electrolyte abnormalities had worse clinical outcomes than those with an eating disorder diagnosis, but no electrolyte abnormalities present (**Table 2**) [29,34,37]. Two studies reported that participants with hyponatremia had a significantly lower lumbar spine BMD z-score (−1.8 ± 1.2 versus −1.3 ± 1.2, P = 0.01) and lower BMI z-score (−2.6 ± 1.3 versus −2.2 ± 1.2, P = 0.04) than compared with those with no documentation of hyponatremia [29,34]. Furthermore, the study by Lawson and colleagues found that a greater proportion of women with hyponatremia reported current psychiatric medication use (83.0%) and amenorrhea (75.0%) than compared to those without hyponatremia (53.0% and 64%, respectively) [29]. Finally, one study assessed outcomes among those with an eating disorder that had an electrolyte abnormity compared to those with an eating disorder, but without electrolyte abnormalities [37]. This work reported that outcomes were more prominent among those with electrolyte abnormalities compared to those without, in domains such as hospitalization (60.5% vs. 47.7%), acute kidney injury (10.4% vs. 3%), chronic kidney disease (12.3% vs. 4.3%), bone fracture (7.0% vs. 4.0%), bowel obstruction (3.6% vs. 1.4%), and mortality (15.7% vs. 5.6%), however there was no association with infection or cardiovascular disease event [37].

### Meta-analyses

**Fig 2:**
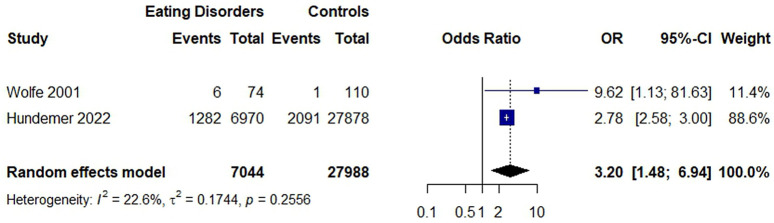
Forest plot of the association between eating disorders electrolyte abnormalities. Cases of eating disorders were compared to healthy controls, free of psychiatric disorders (Wolfe: Sex and weight matched; Hundemer: Sex and age matched).

The first meta-analysis assessed the odds of having an electrolyte abnormality among individuals with an eating disorder diagnosis compared to healthy controls. The pooled odds ratio (OR) from the random effects model indicated that individuals with eating disorders had significantly higher odds of experiencing electrolyte abnormalities compared to controls, with low heterogeneity (OR = 3.20, 95% CI: 1.48–6.94; I^2^ = 22.6%, τ^2^ = 0.1744, p = 0.2556; **Fig 2**).

**Fig 3:**
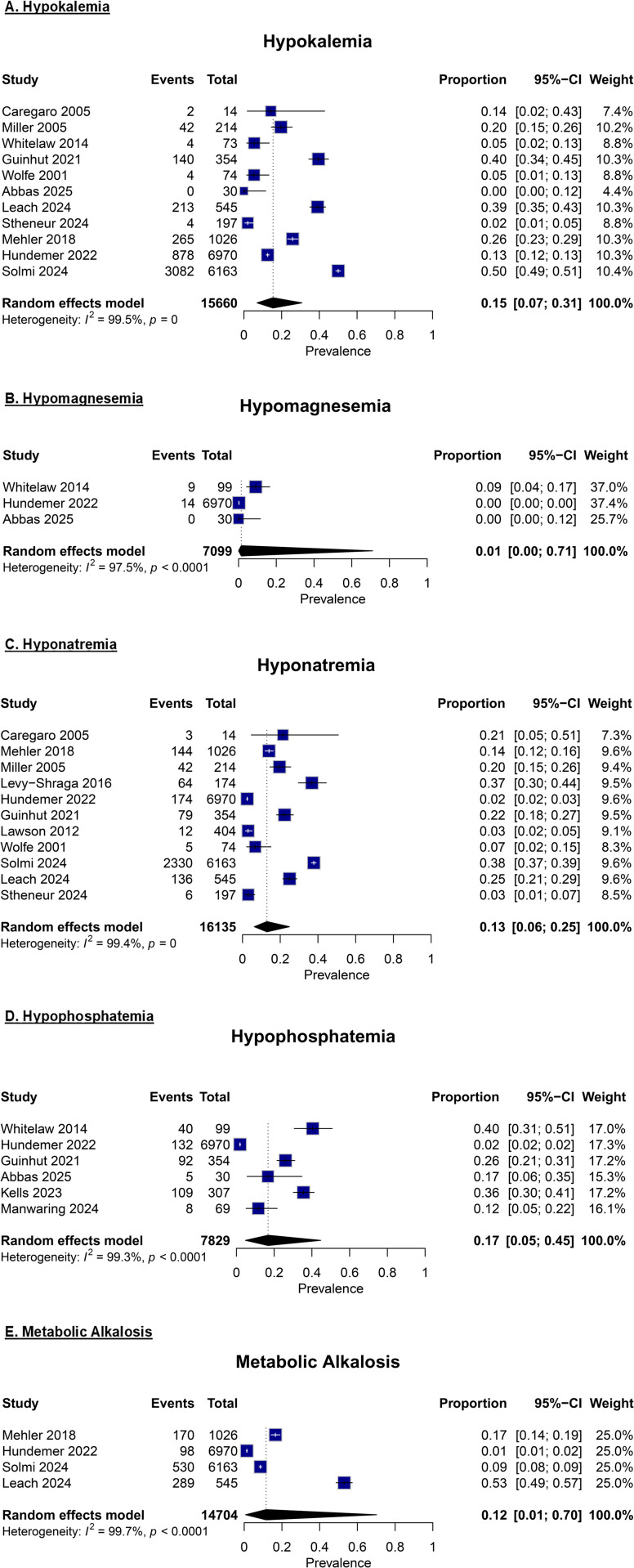
Forest plots assessing the prevalence of (A) hypokalemia, (B) hypomagnesemia, (C) hyponatremia, (D) hypophosphatemia, and (E) metabolic alkalosis.

Next, the prevalence meta-analyses examined electrolyte abnormalities, specifically hypokalemia, hypomagnesemia, hyponatremia, hypophosphatemia, and metabolic alkalosis, among individuals with eating disorders. The pooled prevalence of hypokalemia was 15% (95% CI: 2% – 43%; I^2^ = 99.5%, p = 0; n = 11), hypomagnesemia was 1% (95% CI: 0% – 71%; I^2^ = 97.5%, p < 0.0001; n = 3), hyponatremia was 13% (95% CI: 6% – 25%; I^2^ = 99.4%, p = 0; n = 11), hypophosphatemia was 17% (95% CI: 5% – 45%; I^2^ = 99.3%, p < 0.0001; n = 6), and metabolic alkalosis was 12% (95% CI: 1% – 70%; I^2^ = 99.7%, p < 0.0001; n = 4) (**Fig 3**). All five prevalence estimates showed high heterogeneity across studies.

### Critical appraisal: Methodological quality (JBI)

Utilizing the JBI critical appraisal tool to assess methodological quality, the majority (60%) of the 20 studies included in this review were deemed high quality (S4 Table). The remaining studies (40%) were deemed to be of moderate methodological quality. When analysed by study type, cross-sectional studies possessed the highest methodological quality (100% deemed high quality), followed by case-control (66.6% deemed high quality), and cohort (58.3% deemed high quality).

## Discussion

In this systematic review, comprised of 25,401 individuals with an eating disorder, we found that electrolyte abnormalities were common. In addition, we found that multiple different types of electrolytes were affected and occurred across a broad range of eating disorder types. Few studies reported adverse clinical events directly related to electrolyte abnormalities. Those that did often focused on specific eating disorder diagnoses, such as AN, and particular clinical scenarios, such as hospitalization. The most common adverse outcomes were anemia and bone-related disorders, with few studies reporting overall mortality. Our findings, including the results of the meta-analyses suggest that electrolyte abnormalities are common in individuals with eating disorders (with prevalence ranging based on the type of electrolyte abnormality assessed), and ultimately may be an intermediary to adverse clinical outcomes. The results from our comprehensive analysis, which assessed various types of eating disorders, underscore the importance of vigilant supervision and treatment among patients. Additionally, developing comprehensive treatment plans to address the broader aspects of these conditions is crucial in preventing electrolyte abnormalities and their associated potential adverse outcomes.

Eating disorders are common, predominantly affecting young female populations [47]. In this review, just under half of identified studies restricted their analysis exclusively to female populations. Of those that did include analysis of males, they consistently represented a minority of the study population. However, these findings align with the estimated overall lifetime prevalence of eating disorders, of 8.60% of females and 4.07% of males [48]. Therefore, results and conclusions must be interpreted carefully when generalizing findings across sexes.

Although there is an association identified between a diagnosis of an eating disorder and electrolyte abnormities, there was considerable variation in the prevalence of these abnormalities across studies. This may be attributed to differences in study populations. For instance, some studies included in this review assessed patients admitted to hospital for eating disorder monitoring and treatment [10,30,33–36,38,39,41–46]. It may be assumed that patients being admitted to hospital represent a population of sicker individuals compared to individuals residing in the community or enrolled in outpatient programs. In comparison, studies that assessed health claims data across a larger sample of individuals, on average reported lower proportions of electrolyte abnormalities [27,37,40]. Regardless of the variation in abnormalities identified, studies followed the same trend, reporting higher prevalence of electrolyte abnormalities in individuals with eating disorders. However, one study in this review presented contradictory results. This study by Zeph and colleagues reported that patients with BN had elevated zinc concentrations when compared to healthy age and sex matched controls [38]. One possible explanation for this finding is that individuals with BN may have higher overall nutrient intake, including zinc, during binge episodes, which could contribute to elevated levels [49]. As this finding was a contradictory result, authors called for further large-scale prospective studies on the underlying role of zinc metabolism during various stages of treatment in patients with BN [38]. Furthermore, authors noted the importance of individual nutritional status assessments for clinicians treating patients with eating disorders, as considerable variation in electrolyte abnormalities and adverse clinical outcomes exists [38].

This review compiles evidence on the hypothesis that electrolyte abnormalities may be an intermediary to adverse clinical outcomes, including anemia, skeletal conditions, cardiovascular complications, and mortality. One study included in this review assessed a sample of individuals with an eating disorder diagnosis, analysed according to presence of an electrolyte abnormality [37]. The results showed that individuals with both an eating disorder diagnosis and an electrolyte abnormality had significantly higher risk for several adverse health outcomes including mortality, hospitalization, acute kidney injury, chronic kidney disease, bone fractures, and bowel obstruction, than compared to those with a diagnosis but no electrolyte abnormality present [37]. Other studies have noted how electrolyte abnormalities may be an intermediary to clinical adverse outcomes. Mehler and colleagues hypothesized that hypokalemia may be an early signal of sudden cardiac death, as low serum potassium is a common trigger for structural cardiac abnormalities [10].

Finally, diagnosing and recognizing eating disorders continues to present as a challenge for clinicians. One study included in this review that assessed Medicaid claims data among youth in the state of California found that across the 3-year study period, the majority of the sample (71.4%) did not receive a formal eating disorder diagnosis [40]. Currently, diagnostic criteria rely on measures such as body mass index in AN, and the number of binge-purge episodes in BN, which may vary on a case-by-case basis [50]. As highlighted in this review, the association between electrolyte measures and eating disorder diagnoses may provide objective insight for clinicians, serving as a transdiagnostic marker of severity across eating disorder subtypes.

### Strengths and limitations of this study

A strength of our review is that eligible studies were identified through a comprehensive search of various databases. This search strategy yielded over 3,000 articles for screening. Furthermore, our review is the first to synthesize evidence on the connection between eating disorders and electrolyte abnormalities, and their associated potential adverse outcomes. Additionally, our study included a search of the grey literature, minimizing the potential for publication bias. We also minimized reporting bias by pre-publishing our review protocol. Finally, we assessed the quality of the included studies using a standardized assessment tool, finding that most were of moderate to high quality. Despite its strengths, this systematic review does present some limitations. While we were unable to quantify the association between eating disorders and electrolyte abnormities, we did assess the quality of evidence collected in this review. Using the JBI critical appraisal of methodological quality, we determined that the results presented of this review were of moderate to high quality. Therefore, despite the absence of a meta-analysis, the conclusions drawn from this review remain robust and provide valuable insight in the relationship between eating disorders and electrolyte abnormalities. An additional limitation of this study is potential for residual confounding due to the use of observational data, where unmeasured or inadequately adjusted confounders may still influence the observed associations [51]. Furthermore, few studies considered the temporality in the relationship between electrolyte abnormalities and adverse clinical outcomes. As a result, it makes it difficult to determine whether the electrolyte abnormality is a cause for the clinical outcome. Finally, it is important to acknowledge the inherent challenges when studying eating disorders. Patients with eating disorders are typically not forthcoming with their condition, which contributes to delayed or missed diagnoses [52]. As a result, our understanding of electrolyte abnormalities in the context of eating disorders is not fully understood, which may limit the clinical implications of these findings.

## Conclusion

In conclusion, there is strong evidence of an association between eating disorders and electrolyte abnormalities. Given the various plausible adverse outcomes, our findings reinforce the idea that clinicians must ensure active monitoring of electrolytes and prompt replacement in patients with eating disorders. Furthermore, few studies currently report on the clinical outcomes that may be triggered by eating disorder-related electrolyte abnormalities. Future research should focus on the complexities of these adverse outcomes, the effectiveness of early detection and intervention strategies for electrolyte abnormalities, and their consequences among individuals with eating disorders.

## Supporting information

S1 Table PRISMA 2020 checklist. (DOCX)

S2 Table Detailed medline search strategy. (DOCX)

S3 Table Reasons for study exclusion during full text screening. (DOCX)

S4 Table Joanna briggs institute critical appraisal results for non-randomized controlled trial studies. (DOCX)

## Abbreviations

JBI: Joanna Briggs Institute
PRISMA: Preferred Reporting Items for Systematic Reviews and Meta-Analyses
PROSPERO: , International Prospective Register of Systematic Reviews.
