# Supplementary material for: Eating disorder-related electrolyte abnormalities and adverse outcomes: A systematic review and meta-analysis
Source: PLoS One. 2026 Jun 1;21(6):e0349826. doi: 10.1371/journal.pone.0349826 (PMC13225437; doi:10.1371/journal.pone.0349826)
Supplement: S2 Table — (DOCX) [file pone.0349826.s002.docx]

| **Database** | **Dates** | **Search Strategy** |
| --- | --- | --- |
| Ovid MEDLINE (R) ALL | 1946 to February 2025 | \| # \| Query \| . \| \| --- \| --- \| --- \| \| 1 \| (eating disorders or bulimia nervosa or anorexia nervosa or binge eating disorder).ti,ab. \|  \| \| 2 \| (electrolyte disorders or dehydration or hypokelemia or hyperkalemia or metabolic alkalosis or metabolic acidosis or hyponatremia or hypomagnesemia or hypophosphatemia).ti,ab. \|  \| \| 3 \| 1 and 2 \|  \| \| 4 \| limit 3 to ("adolescent (13 to 18 years)" or "young adult and adult (19-24 and 19-44)" or "middle age (45 to 64 years)" or "all aged (65 and over)") \|  \| \| 5 \| limit 4 to (english or french) \|  \| \| 6 \| *"Hyperphosphatemia"/ \|  \| \| 7 \| "Hyperphosphatemia"/ \|  \| \| 8 \| "feeding and eating disorders"/ or anorexia nervosa/ or avoidant restrictive food intake disorder/ or binge-eating disorder/ or bulimia nervosa/ or food addiction/ \|  \| \| 9 \| (eating disorders or bulimia or anorexia or binge eating disorder).ti,ab. \|  \| \| 10 \| (eating disorders or bulimia or anorexia or binge eating).ti,ab. \|  \| \| 11 \| 8 or 10 \|  \| \| 12 \| water-electrolyte imbalance/ or dehydration/ or hypercalcemia/ or hyperkalemia/ or hypernatremia/ or hypocalcemia/ or hypokalemia/ or hyponatremia/ \|  \| \| 13 \| 2 or 12 \|  \| \| 14 \| 11 and 13 \|  \| \| 15 \| limit 14 to ("adolescent (13 to 18 years)" or "young adult and adult (19-24 and 19-44)" or "middle age (45 to 64 years)" or "all aged (65 and over)") \|  \| \| 16 \| limit 15 to (english or french) \|  \| |
