# Supplementary material for: Eating disorder-related electrolyte abnormalities and adverse outcomes: A systematic review and meta-analysis
Source: PLoS One. 2026 Jun 1;21(6):e0349826. doi: 10.1371/journal.pone.0349826 (PMC13225437; doi:10.1371/journal.pone.0349826)
Supplement: S3 Table — (DOCX) [file pone.0349826.s003.docx]

| **Study (First author, year, title)** | **Reason for Exclusion** |
| --- | --- |
| Nelson 2018: The pitfalls of insufficient clinical reasoning | Ineligible study design |
| Chodorowski 2005: Regression of long standing anorexia nervosa following acute renal failure caused by gentamicin intoxication | Ineligible study design |
| Bahia 2012: Pseudo Bartter syndrome in eating disorders | Ineligible study design |
| Michihata 2010: Elevated cortisol predicts lower phosphorus in prospective study of adolescents hospitalized with anorexia nervosa | Irrelevant context |
| Guerra-Hernandez 2014: Clinical and biochemical findings in Mexican patients with distal renal tubular acidosis | Irrelevant context |
| Kallner 1987: Recurrent factitious hypercalcemia | Published before the year 2000 |
| Woods 1988: Weight control methods in high school wrestlers | Published before the year 2000 |
| Arancibia 2014: A 36 year old man with persistent abdominal pain and nausea | Ineligible study design |
| Casper 1986: The pathophysiology of anorexia nervosa and bulimia nervosa. | Published before the year 2000 |
| Kavak Sinanoglu 2020: A case of Gitelman Syndrome diagnosed with anorexia nervosa in a psychiatry clinic. | Ineligible study design |
| Greenberg 1992: Anorexia nervosa/bulimia and central pontine myelinolysis | Published before the year 2000 |
| Raman 2018: Hypomagnesemia in a Patient With an Eating Disorder | Ineligible study design |
| Davidson 1992: Heart failure secondary to hypomagnesemia in anorexia nervosa | Published before the year 2000 |
| Hardoff 1991: Pathophysiological consequences of eating disorders | Published before the year 2000 |
| Komatsu 2019: Isolated adrenocorticotropic hormone deficiency presenting with severe hyponatremia and rhabdomyolysis: A case report and literature review | Ineligible study design |
| Vladimirov 2020: Central pontine myelinolysis in a patient with bulimia: Case report and literature review | Ineligible study design |
| Ahmed 2008: Chronic Haemolytic Anemia and Splenomegaly in a Patient with an Isolated Adrenocorticotropin Deficiency | Ineligible study design |
| Funayama 2021: Hypokalemia in patients with anorexia nervosa during refeeding is associated with binge-purge behavior, lower body mass index, and hypoalbuminemia. | Irrelevant context |
| Frolich 2016: To the limit of extreme malnutrition | Ineligible study design |
| Lorenzo 2017: Severe hypokalaemia due to diuretic abuse in a teen girl with eating disorder | Ineligible study design |
| Mimura 2021: "Case series: Ischemic stroke associated with dehydration and arteriosclerosis in individuals with severe anorexia nervosa". | Ineligible study design |
| Oden Akman 2019: Sounds unrealistic: an adolescent girl with anorexia nervosa consumes 19 L of fluid in a few hours: what happens to the physiology? | Ineligible study design |
| Golden 2004: Nutritional rehabilitation of anorexia nervosa. Goals and dangers | Ineligible study design |
| Maragh 2011: Appearances can be deceiving: A case of male anorexia | Ineligible study design |
| Freeman 2018: Electrolyte disturbance in eating disorder | Ineligible study design |
| Okabe 1993: Assessment of emaciation in relation to threat to life in anorexia nervosa | Published before the year 2000 |
| Roberts 1986: Unexplained seizure in anorexia nervosa | Published before the year 2000 |
| Schlapfer 2022: Impact of caloric prescriptions and degree of malnutrition on incidence of refeeding syndrome and clinical outcomes in patients with eating disorders: A retrospective review | Irrelevant context |
| Birmingham 1996: Anorexia nervosa: Refeeding and hypophosphatemia | Published before the year 2000 |
| Krogulska 2019: A loss of consciousness in a teenage girl with anorexia nervosa, due to polydipsia: case report and a minireview | Ineligible study design |
| Sinem Akgul 2013: Medical Evaluation of Male Adolescents with an Eating Disorder | Study not available |
| Nagata 2021: Medical complications of eating disorders in boys and men. | Ineligible study design |
| Waddell 2020: Cardiac Arrest and Successful Extracorporeal Cardiopulmonary Resuscitation as a Result of a Refeeding Syndrome in a Young Female with Anorexia Nervosa | Irrelevant context |
| Leroy 2012: Centropontine myelinolysis related to refeeding syndrome in an adolescent suffering from anorexia nervosa | Irrelevant context |
| Baker 1982: Anorexia nervosa. | Published before the year 2000 |
| Lilje 2002: Benign course of central pontine myelinolysis in a patient with anorexia nervosa | Ineligible study design |
| Koda 2020: Vitamin D Deficiency-induced Osteomalacia in a Patient with Anorexia Nervosa | Ineligible study design |
| Bahia 2011: Polydipsia and hyponatremia in a woman with anorexia nervosa | Ineligible study design |
| Raff 2016: Severe anorexia admitted for abdominal pain and failure to thrive | Ineligible study design |
| Kopala 1995: Olfactory identification ability in anorexia nervosa. | Published before the year 2000 |
| Stahl 1998: Prolonged acute renal failure after i.v. immunoglobulin therapy in the refeeding phase of anorexia nervosa | Published before the year 2000 |
| Warren 1979: Acid-base and electrolyte disturbances in anorexia nervosa | Published before the year 2000 |
| Bayir 2016: Anorexia nervosa with fatal hypoglycemic coma | Ineligible study design |
| Koda 2021: Vitamin D deficiency-induced osteomalacia in a patient with anorexia nervosa | Ineligible study design |
| Oden Akman 2020: Sounds unrealistic: an adolescent girl with anorexia nervosa consumes 19 L of fluid in a few hours: what happens to the physiology? | Ineligible study design |
| Kawai 2019: Portal hypertension in prolonged anorexia nervosa with laxative abuse | Ineligible study design |
| Richardson 2021: Retrospective analysis of hypophosphatemia rates and other clinical parameters in patients with eating disorders | Irrelevant context |
| Veronica Gaete 2013: Biomedical Complications and Laboratory Abnormalities at Admission in Young People Attending An Eating Disorder Treatment Program | Study not available |
| Speranza 2021: Nutritional indicators and metabolic alterations in outpatients with anorexia nervosa: a retrospective study | Irrelevant context |
| Wood 2024: Time burden of hospital admissions for severe eating disorders: insights from a district general hospital | Ineligible study design |
| Qazi 2024: Recognition and management of high risk patients with eating disorders in the acute hospital setting: the challenges faced in a district general hospital | Ineligible study design |
| Gordon 2024: Sex Differences in Caloric Needs in Youth With Anorexia Nervosa During Medical Stabilization | Ineligible study design |
| Gaudiani 2012: Severe anorexia nervosa: Outcomes from a medical stabilization unit | Irrelevant context |
| Davies 2017: The medical risks of severe anorexia nervosa during initial re-feeding and medical stabilisation | Irrelevant context |
| Shimizu 2014: Refractory hypoglycemia and subsequent cardiogenic shock in starvation and refeeding: Report of three case | Ineligible study design |
| Frank 2019: Recent advances in understanding anorexia nervosa | Ineligible study design |
| MacIas-Robles 2009: Prolonged QT interval in a man with anorexia nervosa | Ineligible study design |
| Staab 2022: Rapid refeeding in anorexia nervosa: A dialectic balance | Irrelevant context |
| Aamodt 2021: Severe Hypernatremia in an Adolescent With Anorexia Nervosa | Ineligible study design |
| Hall 1988: Refractory hypokalemia secondary to hypomagnesemia in eating-disorder patients | Published before the year 2000 |
| Tachamo 2017: Spontaneous bilateral carpopedal spasm in a bulimic patient | Ineligible study design |
| Eiro 2002: Use of a proton-pump inhibitor for metabolic disturbances associated with anorexia nervosa. | Ineligible study design |
| Leclerc 2013: Evaluation of a nutrition rehabilitation protocol in hospitalized adolescents with restrictive eating disorders | Irrelevant context |
| Willett 2021: Case 38-2021: A 76-Year-Old Woman with Abdominal Pain, Weight Loss, and Memory Impairment | Ineligible study design |
| Franko 1991: Abuse of potassium by a patient with bulimia nervosa | Published before the year 2000 |
| Pascolini 2019: Acute liver injury and anorexia nervosa: A case report | Ineligible study design |
| Brennan 2023: Medical instability in typical and atypical adolescent anorexia nervosa: a systematic review and meta-analysis. | Ineligible study design |
| Katzman 2022: Refeeding Hypophosphatemia in Hospitalized Adolescents with Anorexia Nervosa | Irrelevant context |
| Kishimoto 2014: Tall P waves associated with severe hypokalemia and combined electrolyte depletion. | Ineligible study design |
| Nogal 2008: Evaluation of selected clinical and diagnostic parameters in girls with anorexia nervosa | Irrelevant context |
| Extance 2020: Enteral feeding in patients undergoing curative or neo-adjuvent radiotherapy for oesophageal cancer; is it effective and which patients benefit most? | Irrelevant context |
| Santonastaso 1998: Water intoxication in anorexia nervosa: A case report | Published before the year 2000 |
| Micula-Gondek 2018: Atypical purging behaviors in a patient with anorexia nervosa: consumption of raw red kidney beans as an emetic | Ineligible study design |
| Vootla 2015: Abnormal liver function tests in an anorexia nervosa patient and an atypical manifestation of refeeding syndrome | Irrelevant context |
| Rosen 2016: Liver dysfunction in patients with severe anorexia nervosa | Irrelevant context |
| Meczekalski 2015: Long-term consequences of anorexia nervosa | Ineligible study design |
| Van Uytvanck 2023: Gelatinous bone marrow transformation, pancytopenia, and lung abscess in severe anorexia nervosa | Ineligible study design |
| Gaudiani 2012: Ophthalmic changes in severe anorexia nervosa: A case series | Ineligible study design |
| Hanachi Guidoum 2013: Dysfunctions of liver and intestine in severe malnutrition: A prospective study of evolution after enteral refeeding in patients with anorexia nervosa (AN) | Irrelevant context |
| Di Pascoli 2004: Acute liver damage in anorexia nervosa | Ineligible study design |
| El Ghoch 2016: Management of Severe Rhabdomyolysis and Exercise-Associated Hyponatremia in a Female with Anorexia Nervosa and Excessive Compulsive Exercising | Ineligible study design |
| Tran 2020: Man versus machine? Acquired long QT syndrome in a patient with anorexia nervosa. | Ineligible study design |
| Foppiani 2014: End-stage anorexia nervosa in a young man: multifaceted metabolic, endocrine and infectious derangements managed in an internal medicine setting | Ineligible study design |
| Miller 2016: Re-feeding syndrome and alcoholic cardiomyopathy: A case of interacting diagnoses | Ineligible study design |
| Ekart 2012: Rare manifestation of the TTP/HUS in a single patient with sarcoidosis, common variable immunodeficiency and lymphoma | Ineligible study design |
| De Alves Pereira Carvalho Saraiva 2022: Pseudo Bartter Syndrome in anorexia nervosa | Ineligible study design |
| Lai 2017: Manifestation of hyperaldosteronism related hypokalemia in a case of anorexia nervosa | Ineligible study design |
| Katzman 2014: Refeeding hypophosphatemia in hospitalized adolescents with anorexia nervosa: A position statement of the society for adolescent health and medicine | Irrelevant context |
| Braude 2018: Refeeding electrolyte derangement in an adult inpatient population with anorexia nervosa: A retrospective analysis | Irrelevant context |
| Gibson 2020: Extreme anorexia nervosa: Medical findings, outcomes, and inferences from a retrospective cohort. | Ineligible study design |
| Raj 2012: Hypomagnesemia in adolescents with eating disorders hospitalized for medical instability | Irrelevant context |
| Hughes 2020: Hypokalemia: A Curious Case in a Young Woman | Ineligible study design |
| Lee 2013: Gitelman's syndrome with vomiting manifested by severe metabolic alkalosis and progressive renal insufficiency | Ineligible study design |
| Wang 2016: Male anorexia nervosa: A unifying diagnosis for multisytemic symptoms | Ineligible study design |
| Deng 2019: Severe hypophosphatemia in a patient with a relapsing lymphoma | Ineligible study design |
| Anonymous 2014: Refeeding hypophosphatemia in hospitalized adolescents with anorexia nervosa: a position statement of the Society for Adolescent Health and Medicine. | Irrelevant context |
| Palm 2016: Kwashiorkor: An unexpected complication to anorexia nervosa | Ineligible study design |
| Harewood 2017: Air hunger-a case report of spontaneous asymptomatic pneumothorax and pneumomediastinum in male with severe anorexia | Ineligible study design |
| Wada 2008: A case report of an anorexia nervosa patient with end-stage renal disease due to pseudo Bartter's syndrome and Chinese herb nephropathy requiring maintenance hemodialysis | Ineligible study design |
| Iacopelli 2022: Delayed appearance of refeeding syndrome in a patient with anorexia nervosa: A case report | Irrelevant context |
| Bando 2005: Central pontine myelinolysis associated with a hypoglycemic coma in anorexia nervosa. | Ineligible study design |
| Eiro 2002: Use of a proton-pump inhibitor for metabolic disturbances associated with anorexia nervosa | Ineligible study design |
| Zimmermann-Viehoff 2007: Acute endocarditis in a patient with severe anorexia nervosa and autoaggressive behavior. | Ineligible study design |
| Crnkovic 2016: Acute metabolic complications and nutritional assesment in children and adolescents with anorexia nervosa | Irrelevant context |
| Schretlen 2022: An Unexpected Cause of Hypokalemia | Ineligible study design |
| Khoury 2022: Hungry Hungry Bones: Electrolyte Abnormalities in the Presence of Severe Malnutrition | Ineligible study design |
| Lindblade 2005: Anorexia Nervosa and Ventricular Fibrillation | Ineligible study design |
| Huang 2020: Normotensive hypokalemic primary hyperaldosteronism mimicking clinical features of anorexia nervosa in a young patient: A case report | Ineligible study design |
| Kells 2022: Response to "Refeeding hypophosphatemia in adolescents with anorexia nervosa" | Ineligible study design |
| Lim 2008: Anorexia nervosa and senna misuse: Nephrocalcinosis, digital clubbing and hypertrophic osteoarthropathy | Ineligible study design |
| Kara 2013: Psychogenic polydipsia in an adolescent with eating disorder: a case report | Ineligible study design |
| Parkash 2014: Refeeding syndrome in a young girl with anorexia nervosa | Ineligible study design |
| Chuang 2018: Reducing electrolyte monitoring for refeeding syndrome in adolescents with disordered eating | Irrelevant context |
| Rastogi 2016: Unusual cause of profound weight loss in a young woman | Ineligible study design |
| Martin 2018: Bulimia nervosa-the silent killer-a case report from paediatric intensive care | Ineligible study design |
| Pehlivanturk-Kizilkan 2020: Refeeding Hypophosphatemia Risk in Adolescent Inpatients with Anorexia Nervosa During Nutritional Rehabilitation | Irrelevant context |
| Swenne 2000: Heart risk associated with weight loss in anorexia nervosa and eating disorders: Electrocardiographic changes during the early phase of refeeding | Irrelevant context |
| Karahmadi 2011: High level increase in liver enzymes and severe thrombocytopenia in a male case of anorexia nervosa | Ineligible study design |
| Das 2019: An unusual presentation of anorexia nervosa as cardiac failure in an adolescent girl | Ineligible study design |
| Curran 2017: An Unusual Etiology of Hypokalemia in a Patient With an Eating Disorder | Ineligible study design |
| Fisher 2000: Hypophosphatemia secondary to oral refeeding in anorexia nervosa | Irrelevant context |
| Gaudiani 2016: Medical outcomes for adults hospitalized with severe anorexia nervosa: An analysis by age group | Irrelevant context |
| Tazoe 2007: Hyperkalemia and hyperdopaminemia induced by an obsessive eating of banana in an anorexia nervosa adolescent | Ineligible study design |
| Luthra 2004: Anorexia nervosa and chronic renal insufficiency: A prescription for disaster | Ineligible study design |
| Pavletic 2011: Hyperkalemia Induced by Excessive Consumption of Dried Fruits-Manifestation of an Undiagnosed Eating Disorder? | Ineligible study design |
| Chu 2012: ACUTE center for eating disorders | Ineligible study design |
| Margrat 2022: A Case Report on Anorexia Nervosa | Ineligible study design |
| Draffin 2022: Comparison of a low carbohydrate intake and standard carbohydrate intake on refeeding hypophosphatemia in children and adolescents with anorexia nervosa: a pilot randomised controlled trial. | Irrelevant context |
| Kells 2022: Factors associated with refeeding hypophosphatemia in adolescents and young adults hospitalized with anorexia nervosa | Irrelevant context |
| Bamhraz 2021: Diagnostic Dilemma in an Adolescent Girl with an Eating Disorder, Intellectual Disability, and Hypomagnesemia | Ineligible study design |
| Nickel 2019: Altered cortical folding and reduced sulcal depth in adults with anorexia nervosa | Irrelevant context |
| Kleisoura 2018: Acute retropharyngeal abscess in a patient with anorexia nervosa | Ineligible study design |
| LiCavoli 2011: Acute kidney injury in a patient with psychological eating disorder | Ineligible study design |
| Kells 2019: Factors Associated With Refeeding Hypophosphatemia (RH) In Hospitalized Adolescents And Young Adults (AYA) With Anorexia Nervosa (AN) | Irrelevant context |
| Steckler 1995: Central pontine myelinolysis in a patient with bulimia | Published before the year 2000 |
| Mehler 2003: Bulimia nervosa | Ineligible study design |
| Munoz 2002: Anorexia nervosa in female adolescents: Endocrine and bone mineral density disturbances | Ineligible study design |
| Bolhuis 2024: Adherence to Dutch Guideline in Hospitalized Anorexia Nervosa Adolescents | Irrelevant context |
| Codesal 2015: Eating disorders: A diagnosis to consider in male population. A case report and review of current data | Ineligible study design |
| Abed 2014: "fixing a heart": The game of electrolytes in anorexia nervosa | Ineligible study design |
| Krogulska 2020: "A loss of consciousness in a teenage girl with anorexia nervosa, due to polydipsia: Case report and a minireview": Correction. | Ineligible study design |
| Azumagawa 2007: Anorexia nervosa and refeeding syndrome. A case report. | Irrelevant context |
| Huang 2001: Life-threatening refeeding syndrome in a severely malnourished anorexia nervosa patient | Irrelevant context |
| Sugimoto 2003: Central pontine myelinolysis associated with hypokalaemia in anorexia nervosa | Ineligible study design |
| Takaya 2008: Surged leptin/ghrelin secretion associated with anorexia nervosa | Ineligible study design |
| Olson 2005: Outpatient management of electrolyte imbalances associated with anorexia nervosa and bulimia nervosa. | Ineligible study design |
| Rome 2003: Medical complications of eating disorders: An update | Ineligible study design |
| Gentile 2013: Metabolic and nutritional needs to normalize body mass index by doubling the admission body weight in severe anorexia nervosa | Ineligible study design |
| Bang 2021: Associations of age, body mass index and biochemical parameters with brain morphology in patients with anorexia nervosa | Irrelevant context |
| Boto 2019: Cerebral Gray and White Matter Involvement in Anorexia Nervosa Evaluated by T1, T2, and T2* Mapping | Irrelevant context |
| Evrard 2004: Impaired osmoregulation in anorexia nervosa: A case-control study | Irrelevant context |
| Rosen 2023: Renal fluid and acid/base balance during refeeding in restrictive eating disorders | Irrelevant context |
| Suwabe 2005: Marked ascites and serum carbohydrate antigen (CA) 125 elevation in a patient with anorexia nervosa | Ineligible study design |
| Monti 2003: [Hypophosphatemia and refeeding syndrome: a severe and underdiagnosed adverse effect]. | Irrelevant context |
| Birmingham 2004: Hypomagnesemia during refeeding in anorexia nervosa | Irrelevant context |
| Al-Habeeb 2005: Anorexia nervosa: Emphasis on its medical complications | Ineligible study design |
| Whitelaw 2018: Predictors of Complications in Anorexia Nervosa and Atypical Anorexia Nervosa: Degree of Underweight or Extent and Recency of Weight Loss? | Irrelevant context |
| Matsunaga 2024: Severe hypoglycemia with reduced liver volume as an indicator of end-stage malnutrition in patients with anorexia nervosa: A retrospective observational study. | Irrelevant context |
| Gaudiani 2014: Low prealbumin is a significant predictor of medical complications in severe anorexia nervosa | Irrelevant context |
| Rome 2012: Eating disorders in children and adolescents | Ineligible study design |
| Loisel 2022: When refeeding is not enough: severe and prolonged pancytopenia in an adolescent with anorexia nervosa | Ineligible study design |
| Amann 2001: Central pontine myelinolysis in a patient with anorexia nervosa. | Ineligible study design |
| O'Neil 2021: A Case of Spontaneous Coronary Artery Dissection in a Patient with Severe Anorexia Nervosa | Ineligible study design |
| Copeland 1989: Diuretic abuse and central pontine myelinolysis | Ineligible study design |
| Leitner 2016: Prophylactic Phosphate Supplementation for the Inpatient Treatment of Restrictive Eating Disorders | Irrelevant context |
| Maiolo 2017: Severe hypokalaemia due to diuretic abuse in a teen girl with eating disorder | Ineligible study design |
| Redgrave 2015: Refeeding and weight restoration outcomes in anorexia nervosa: Challenging current guidelines | Irrelevant context |
| Sekot 2009: Refeeding syndrome and central pontine myelinolysis in the patient with anorexia nervosa | Irrelevant context |
| Kular 2013: So basic! | Ineligible study design |
| Hanachi 2020: Echocardiographic abnormalities in 124 severely malnourished adult anorexia nervosa patients: Frequency and relationship with body composition and biological features. | Irrelevant context |
| Ensminger 2003: 35-Year-old woman with cough, fever, and anorexia | Ineligible study design |
| Hunter 2018: An unusual case of abdominal pain and hyponatremia in a 16-year-old girl with disordered eating | Ineligible study design |
| Weiss 2014: Conservative inpatient refeeding yields modest outcomes in adolescents with anorexia nervosa and eating disorder not otherwise specified | Irrelevant context |
| Bridet 2014: Acute liver damage and anorexia nervosa: A case report | Ineligible study design |
| Chadi 2017: Nephrocalcinosis in a young male with anorexia nervosa | Ineligible study design |
| Koga 2019: Portal hypertension in prolonged anorexia nervosa with laxative abuse: A case report of three patients | Ineligible study design |
| Cereda 2016: Myocardial changes in patients with anorexia nervosa | Ineligible study design |
| Nakahara 2006: The effects of bone therapy on tibial bone loss in young women with anorexia nervosa | Ineligible study design |
| Saito 2014: Management and treatment of eating disorders with severe medical complications on a psychiatric ward: A study of 9 inpatients in Japan | Ineligible study design |
| Lamzabi 2015: Myocardial changes in a patient with anorexia nervosa: A case report and review of literature | Ineligible study design |
| DeJager 2011: Metabolic and neurologic sequelae in a patient with long-standing anorexia nervosa who presented with septic shock and deep hypoglycemia | Ineligible study design |
| Roy-Lavallee 2020: Scurvy: An Unexpected Nutritional Complication in an Adolescent Female With Anorexia Nervosa | Ineligible study design |
| Fotheringham 2005: Refeeding syndrome: Life-threatening, underdiagnosed, but treatable | Irrelevant context |
| Boyd 2018: Recurrent hyponatremia in a young adult woman with anorexia nervosa and the effects of insufficient communication | Ineligible study design |
| Park 2011: Orthorexia nervosa with hyponatremia, subcutaneous emphysema, pneumomediastinum, pneumothorax, and pancytopenia | Ineligible study design |
| Volman 2011: Tako Tsubo cardiomyopathy, presenting with cardiogenic shock in a 24-year-old patient with anorexia nervosa | Ineligible study design |
| Whitelaw 2009: Does aggressive refeeding in hospitalised adolescents with anorexia nervosa result in increased hypophosphataemia? | Irrelevant context |
| Golden 2013: Higher caloric intake in hospitalized adolescents with anorexia nervosa is associated with reduced length of stay and no increased rate of refeeding syndrome | Irrelevant context |
| Funayama 2021: Body mass index and blood urea nitrogen to creatinine ratio predicts refeeding hypophosphatemia of anorexia nervosa patients with severe malnutrition. | Irrelevant context |
| Sung 2015: Etiologic and therapeutic analysis in patients with hypokalemic nonperiodic paralysis. | Irrelevant context |
| Chau 2005: Bulimia nervosa presenting as acute cardiogenic shock | Ineligible study design |
| El Midaoui Aouatef 2010: Anorexia nervosa associated with central pontine myelinolysis and extrapontine myelinolysis | Ineligible study design |
| Kawada 2022: Refeeding hypophosphatemia in adolescent inpatients with anorexia nervosa | Irrelevant context |
| Weiner 2012: Severe hypernatremia in an adolescent with an eating disorder | Ineligible study design |
| Biffl 2010: The management of pneumothorax in patients with anorexia nervosa: A case report and review of the literature | Ineligible study design |
| Wassenaar 2018: A causality dilemma: ARFID, malnutrition, psychosis, and hypomagnesemia | Ineligible study design |
| Nagata 2024: Sex differences in electrolyte abnormalities indicating refeeding syndrome risk among hospitalized adolescents and young adults with eating disorders. | Irrelevant context |
| Khatri 2023: A Multi-Disciplinary Approach to Managing End-Stage Renal Disease in Anorexia Nervosa: A Case Report | Ineligible study design |
| Neychev 2015: Bowel ischemia and necrosis in anorexia nervosa: A case report and review of the literature | Ineligible study design |
| Curran 2016: Case report: An unusual etiology of hypokalemia in a patient with an eating disorder | Ineligible study design |
| Krogulska 2019: "A loss of consciousness in a teenage girl with anorexia nervosa, due to polydipsia: Case report and a minireview": Correction. | Ineligible study design |
| Taube 2021: Hyponatremia caused by water intoxication: Successful treatment of psychiatric disturbances with olanzapine and fluoxetine | Ineligible study design |
| Foppiani 2017: Near-fatal anorexia nervosa in a middle-aged woman | Ineligible study design |
| Hershkowitz 2015: Thiamine deficiency in self-induced refeeding syndrome, an undetected and potentially lethal condition | Irrelevant context |
| Hancher 2020: Severe hypernatremia and gastric dilation from chronic eating disorder and intentional salt ingestion | Ineligible study design |
| Raj 2012: Hypomagnesemia in adolescents with eating disorders hospitalized for medical instability | Irrelevant context |
